# Physicochemical Properties of Hom Nil (*Oryza sativa*) Rice Flour as Gluten Free Ingredient in Bread

**DOI:** 10.3390/foods7100159

**Published:** 2018-09-27

**Authors:** Lalana Thiranusornkij, Parichart Thamnarathip, Achara Chandrachai, Daris Kuakpetoon, Sirichai Adisakwattana

**Affiliations:** 1 Technopreneurship and Innovation Management Program, Graduate School, Chulalongkorn University, Bangkok 10330, Thailand; Lalana@kcgcorporation.com (L.T.); Chandrachai@yahoo.com (A.C.); 2 KCG Excellence Center, KCG Corporation Co., Ltd., Thepharak Rd., Bangpleeyai, Bangplee, Samutprakarn 10540, Thailand; Parichart.t@kcgcorporation.com; 3 Department of Food Technology, Faculty of Science, Chulalongkorn University, Bangkok 10330, Thailand; Kdaris@gmail.com; 4 Department of Nutrition and Dietetics, Faculty of Allied Health Sciences, Chulalongkorn University, Bangkok 10330, Thailand

**Keywords:** Hom Nil, Hom Mali, physicochemical properties, antioxidant, gluten free, bread

## Abstract

Hom Nil (*Oryza sativa*), a Thai black rice, contains polyphenolic compounds which have antioxidant properties. The objective of this study was to investigate physicochemical properties of Hom Nil rice flour (HN) and its application in gluten free bread by using Hom Mali 105 rice flour (HM) as the reference. The results demonstrated that HN flour had significantly higher average particle sizes (150 ± 0.58 µm), whereas the content of amylose (17.6 ± 0.2%) was lower than HM flour (particle sizes = 140 ± 0.58 µm; amylose content = 21.3 ± 0.6%). Furthermore, HN contained higher total phenolic compounds (TPC) (2.68 ± 0.2 mg GAE/g flour), total anthocyanins (293 ± 30 mg cyanidin-3-glucoside/g flour), and the ferric reducing antioxidant power (FRAP) (73.5 ± 1.5 mM FeSO_4_/g) than HM flour (TPC = 0.15 mg GAE/g flour and FRAP = 2.24 mM FeSO_4_/g flour). In thermal properties, the onset temperature (T_o_), the peak temperature (T_p_) and the conclusion (T_c_) temperature of HN flour were similar to the values of HM flour. However, HN flour had lower enthalpy change (ΔH) than HM flour. The results showed that HN flour had lower swelling power and higher solubility than HM flour at the temperature between 55 °C and 95 °C. In pasting properties, HN flour also showed lower peak, trough and breakdown viscosity than HM flour. In addition, the bread samples prepared by HN flour had higher value of hardness and lower value of cohesiveness than the bread prepared from HM flour. Taken together, the findings suggest that HN flour could be used as an alternative gluten-free ingredient for bread product.

## 1. Introduction

Carbohydrates are a major macronutrient of energy intake in the human diet. It has been reported that overconsumption of dietary carbohydrate has been associated with increased risks of obesity and metabolic diseases [1]. Rice is widely consumed as a staple food for many people, especially those living in Asia. Khaw Dok Mali 105 or Hom Mali (HM) is one of the most popular varieties of white rice that has a slightly floral aroma and a soft, sticky texture when cooked [2]. However, white rice flour obtained after polishing and milling lost its nutrition values including ash, protein, fat and fiber which were mostly contained in the outer layer [3]. Recently, dark-colored rice has emerged as a potential functional ingredient because of the presence of nutrients and phytochemical compounds including anthocyanins, flavones, flavanols, carotenoids and γ-oryzanols [4]. Brown and white rice contained low amounts of flavones and γ-oryzanols [4]. Interestingly, pigmented rice is a rich source of phytochemicals responsible for positive potential effects on human health [4]. It has been shown that the fortification of anthocyanin-rich black rice extract powder (≥89.6% cyanidin-3-glucoside) at certain content (1–2% *w/w* of flour) in wheat bread was effective to reduce the digestion rate of starch and provided bread with comparable quality [5]. The combination of purple rice flour and wheat flour improved important nutritional properties, causing the reduction of predicted glycemic index and increased antioxidant property [6]. In food application, the pigmented rice flour is considered as a potential bioactive ingredient for functional foods. For example, riceberry rice flour, a dark purple rice flour was used to replace wheat for nutritional purpose in noodle [7]. In addition, black glutinous rice flour was able to prepare cake with 100% substitution into wheat [8]. Black rice powder could be added up to 5% into pork patty with acceptable sensory qualities [9].

Hom Nil (*Oryza sativa*), a Thai black rice, has been reported as a good source of antioxidants, which contains phenolic compounds [10]. Previous studies have demonstrated the biological properties of Hom Nil rice in various experimental models. For example, the extract from Hom Nil rice showed antimutagenicity against mutagens (nitrite-treated 1-aminopyrene and nitrite treated chicken essence) [11]. It suggested that the active compounds in Hom Nil rice have a potential to inhibit the mutagenicity of compounds obtained from the interaction between nitrite ion, particularly of fermented meat products, and some convertible compounds of food during the acidic conditions of stomach digestion [11]. The polyphenolics in Hom Nil could inhibit the formation of advanced glycation end products (AGEs) [12]. The components in rice bran of pigmented rice such as Hom Nil are polyphenolics, anthocyanins and others (γ-oryzanol, α-tocopherol, and ferulic acid) which have antioxidant and anti-inflammation activity in human promyelocytic leukemia (HL-60) cell [13]. In addition, Hom Nil rice also contains β-carotene considered as an effective quencher of singlet oxygen by reaction with peroxyl radicals, resulting in inhibition of propagation and termination of oxidation chain reactions [14]. Although Hom Nil rice presented promising bioactivity in vitro and in vivo, its physicochemical properties of Hom Nil and its potential food application in flour-based products remain unknown. The objectives of this study were to investigate physicochemical properties of Hom Nil and its application in gluten free bread in comparison with Hom Mali rice flour.

## 2. Materials and Methods

### 2.1. Chemicals and Reagents

Hom Nil rice and Khaw Dok Mali 105 (Hom Mali 105 rice) were randomly purchased from a local farmer community of Yasothorn Province, Thailand during May–June 2016. Each batch of the sample was prepared by pooling and mixing of the three different areas of purchasing. Folin & Ciocalteu’s phenol reagent, gallic acid, 2,4,6-Tri (2-pyridyl)-s-triazine (TPTZ), amylose from potato and other chemical reagents were purchased from Sigma-Aldrich (St. Louis, MO, USA).

### 2.2. Flour Preparation

Rice flour was ground using dry-milling method with an Alpine Pin Mill (Phoenix Equipment Corporation, Red Bank, NJ, USA) with voltage 50 Hz and speed 4800 rpm. The percent yield of Hom Nil (HN) and Hom Mali 105 (HM) flour was 97.5%. The rice flour was screened into particle sizes of 125–180 µm using sieve of 125 µm and 180 µm sizes. The flour was stored in sealed ethylene-vinyl alcohol copolymer (EVOH) bags at −20 °C until further analysis. The moisture contents of HN and HM and RB were 11.9% and 12.9%, respectively.

### 2.3. Analysis of Particle Size Distributions

The particle size distributions of flour were measured using a Laser particle size analyzer (Mastersizer 3000, Malvern Instrument Ltd., Worcestershire, UK) with dry dispersion module according to Kraithong et al. [3]. The measurement was performed under vacuum with 2 bars of pressure and 50% of feed rate. The cumulative weight percent particle size plots and the mean particle sizes were calculated by the instrument’s software. 

### 2.4. Determination of Amylose Content

The content of amylose in rice flour was determined according to a modified method of AACC [15]. Rice flour (100 mg) was mixed with 95% ethanol (1 mL) and 1 N NaOH (9 mL) and left to stand at room temperature for 10 min then heated for 10 min in a water bath (100 °C) and cooled to room temperature for at least 2 h. The solution was made up to 100 mL with distilled water and vortexed vigorously. The sample solution (5 mL) was incubated with 50 mL distilled water, 1 N acetic acid (2 mL) and iodine solution (2 mL of 0.2 g iodine and 2.0 g potassium iodide in 100 mL of aqueous solution) and then adjusted to final volume of 100 mL with distilled water. The absorbance was read at 620 nm after standing for 20 min. Amylose content was determined using a standard curve of pure amylose from potato.

### 2.5. Determination of Total Phenolic and Anthocyanin

The extraction was performed according to the modified method of Shen et al. [16]. In brief, milled rice powder (50 g) was soaked in 200 mL methanol with 1.5% HCl overnight at room temperature and shaken at 150 rpm. The extract was filtrated through filter paper Whatman No.1. The extract was evaporated until dried and then stored at −20 °C until analysis. The total phenolic content of extracts was performed according to a modified method of Adisakwattana et al. [17]. The extract was freshly dissolved in 80% methanol prior to use. Briefly, 10 μL of sample solution (1.0 mg/mL) was incubated with 100 μL of Folin–Ciocalteu’s reagent (10-fold dilution in distilled water before use for 5 min). After incubation, 80 μL of 1 M sodium carbonate solution was added and incubated for 30 min at room temperature. The absorbance was measured at 760 nm. The content of total anthocyanin in Hom Nil rice flour (1.0 mg/mL extract) was measured using a spectrophotometric pH-differential method according to the previous method [18]. The extract was added to two buffer systems including 0.025 M potassium chloride at pH 1.0 and 0.4 M sodium acetate at pH 4.5 respectively. The calculated absorption was determined using the equation of A = (A_λ510_ − A_λ700_) pH 1.0 − (A_λ510_ − A_λ700_) pH 4.5 and total monomeric anthocyanins were expressed as cyanidin-3-glucoside.

### 2.6. Ferric Reducing Antioxidant Power Assay

The assay of ferric reducing antioxidant power (FRAP) of flour was determined according to Benzie; Strain [19] with minor modifications. The rice flour extract was diluted to 1:5 with 0.1 M phosphate buffer saline (pH 7.4). The FRAP reagent contained 0.3 M of sodium acetate buffer saline (pH 3.6), 10 mM TPTZ in 40 mM HCl and 20 mM FeCl_3_ 90 µL of FRAP reagent was mixed with 10 µL of the rice extract at a ratio of 10:1:1. The reaction mixture was incubated in the dark at room temperature for 30 min and the absorbance was read at 595 nm. The FRAP value was calculated from the calibration curve of FeSO_4_. 

### 2.7. Thermal Properties

The measurement of differential scanning calorimetry (DSC) for degree of gelatinization on rice flour samples was performed using a differential scanning calorimeter (Netzsch DSC 204F1 Phoenix^®^, Selb, Germany) according to a previous study [20]. The rice flour sample (3 mg, dry basis) was precisely weighed and mixed with deionized distilled water (10 µL) and put into sample pans. The mixture was sealed in the pan at room temperature for 1 h. The samples were heated in a temperature range of 25–100 °C with a heating rate of 10 °C/min. An empty aluminum pan was used as a reference. 

### 2.8. Swelling Power and Water Solubility

The sample (0.25 g) was heated in 10 mL distilled water in a water bath at 55, 65, 75, 85, and 95 °C for 30 min with constant mixing and then cooled to room temperature. The samples were centrifuged at 2000 rpm for 20 min. The precipitated and the supernatant part were dried at 105 °C and weighed in order to calculate swelling power and water solubility of rice flour according to the modified method of Leach et al. [21].

### 2.9. Pasting Properties

The pasting property of rice flour (3 g flour based on 14% moisture in 25 g distilled water) was determined using a Rapid Visco Analyser (RVA 4500 Newport Scientific, MN, USA) according to a previous study [22]. The flour suspension was heated to 50 °C for 1 min and then heated to 95 °C at rate of 12 °C/min. The sample was kept at 95 °C for 2–3 min before cooling to 50 °C at the rate of 12 °C/min. Finally, it was constantly stirred at a speed of 960 rpm, and the total run time was 13 min. The pasting properties were expressed as peak viscosity, trough, breakdown, final viscosity, setback, peak time and pasting temperature.

### 2.10. Bread Preparation

The rice bread from HN and HM flour was prepared according to a previous study [23]. The following ingredients based on 100 g rice flour were used for bread making; sugar (5%), salt (1.8%), yeast (3%), Benecel (methylcellulose and hydroxypropyl methylcellulose) (2%) (Ashland, Covington, KY, USA) and were mixed using a Kitchen-Aid bowl mixer with a C-dough hook at speed 53 rpm for 1 min. Vegetable oil (6%), white egg (40%) and milk (70%) were added and then mixed together at speed of 160 rpm for 10 min. After that, batter was poured into a mold, placed at 30 °C and 90% relative humidity for 50 min and baked at 150 °C for 40 min. The loaf was removed from the mold and cooled at room temperature. The bread sample was packed in a sealed polyethylene bag until analysis. 

### 2.11. Texture Properties of Bread

Texture analysis was measured according to a modified method of Kang et al. [24] using a texture analyzer (Lloyd Instruments, Fareham, UK) in combination with a 5 kg load cell fitted with a 50 mm diameter cylinder aluminum probe at a constant speed of 1.0 mm/s with strain rate of 50%. Bread crumbs were prepared as 25 × 25 × 25 mm slabs. The hardness, cohesiveness, chewiness, springiness and adhesiveness were measured from the texture profile analysis. Hardness is the force required to compress the material indicating degree of force required at first bite [25]. The cohesiveness shows the internal resistance or cohesion of food structure [23]. Chewiness is defined as the energy required to masticate a solid food product [26]. Springiness value relates to the elasticity of bread [22]. Adhesiveness is the energy required to overcome attractive force between the food and any surface it is in contact. Specific volume of bread was measured by the seed displacement method [27]. The values were reported as the ratio between the volume of the bread and its weight in cm^3^/g.

### 2.12. Statistical Analysis

Data were expressed as mean ± standard error of mean (SEM) with three or five independent batches, in which duplicates were tested for each assay. The statistical significance of the results was evaluated using an independent t-test to analyze the mean and the differences between the means. The analysis of variance carried out with Duncan’s multiple test was performed to conduct significant difference for swelling power and solubility. The value of *p* < 0.01 was considered statistically significant.

## 3. Results and Discussion

### 3.1. Particle Size Distribution and Amylose Content of Rice Flour

**Figure 1:**
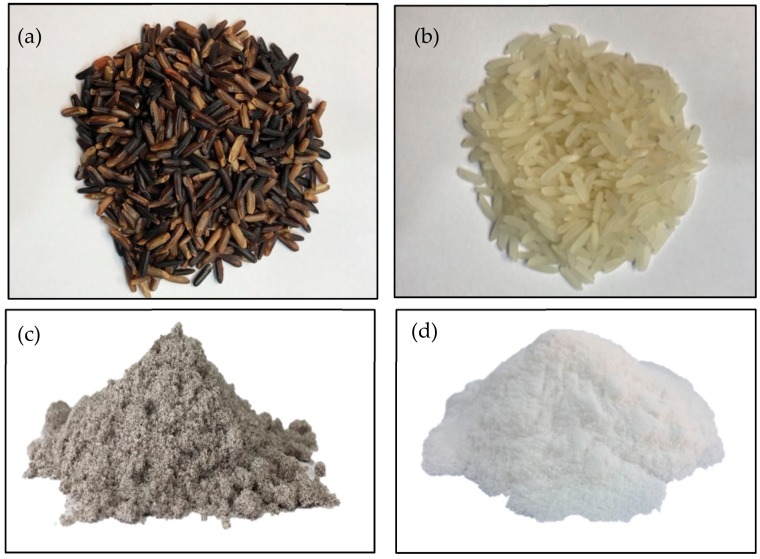
The appearance of Hom Nil rice (HN) (**a**) and Hom Mali 105 rice (HM) (**b**), HN flour (**c**) and HM flour (**d**).

As shown in Figure 1, HM and its flour are white particle, whereas HN and its flour are reddish purple particle. 

**Figure 2:**
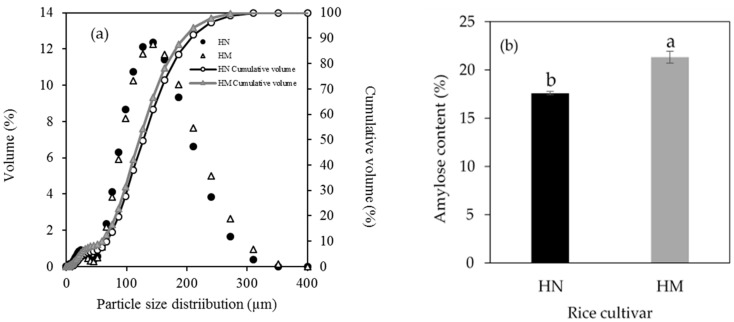
Particle size distribution (**a**) and amylose content (**b**) of Hom Nil rice flour (HN) and Hom Mali 105 rice flour (HM). The results are expressed as mean ± standard error of the mean (SEM), *n* = 3. The different letters denote statistically significant differences in mean values (*p* < 0.01).

Previous studies demonstrated that the large particle size of rice flour (100–200 µm) provided preferable bread properties with less hardness and high specific volume [23,28,29]. Therefore, the particle size of rice flour ranging from 125 to 180 µm were chosen for this study. The curve of particle size distribution of rice flour is represented in Figure 2a. The bimodal particle sizes of rice flour were observed. The average particle size of HN and HM which was 150 ± 0.58 µm and 140 ± 0.58 µm, respectively. The results showed that HN had significantly higher particle size than HM. The different particle size of rice flour is related to cultivar and method of milling, resulting in the differences in granule composition [30]. The results, in agreement with a study of Drakos et al. indicate that different particle diameters of barley and rye flours are observed when using different method of milling process [31]. In addition, the results showed that HN flour demonstrated higher particle size than HM flour. It suggests that HN rice does not undergo a polishing process, it retains part of the bran, leading to the presence of the large particle size of HN flour after milling. The large particle size of flour contributes to its functional properties including starch gelatinization and pasting properties [32]. 

The amylose content of HN was significantly lower than HM (Figure 2b). According to categorizing rice based on the amylose content [33], HN and HM were considered as low amylose (12–20%) and medium amylose (20–25%), respectively. The amylose content of HN and HM were in the same range as previously studied [34,35,36]. It suggests that the difference in amylose content between HN and HM is partly due to their cultivar varieties [37,38]. Moreover, the report demonstrated that texture of cooked rice is affected by amylose content [39].

### 3.2. Phytochemical Compounds and Antioxidant Activity of Rice Flour

**Figure 3:**
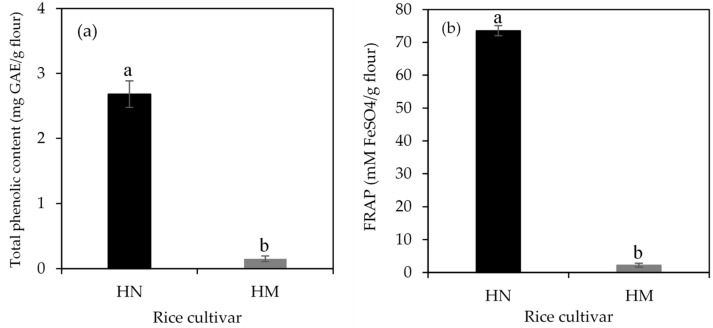
Total phenolic content (**a**) and ferric reducing antioxidant power (FRAP) (**b**) of Hom Nil rice flour (HN) and Hom Mali 105 rice flour (HM). The results are expressed as mean ± standard error of the mean (SEM), *n* = 3. The different letters denote statistically significant differences in mean values (*p* < 0.01).

Total phenolic content (TPC) and ferric reducing antioxidant power (FRAP) are shown in Figure 3a,b, respectively. TPC and FRAP were found in HN and HM while the content of anthocyanin (ACN) could not detected in HM. The higher values of TPC, FRAP and ACN observed in the HN flour were 2.68 ± 0.2 mg GAE/g flour, 73.5 ± 1.5 mM FeSO_4_/g flour and 293 ± 30 mg cyanidin-3-glucoside/g flour, compared to those in the HM flour (TPC = 0.15 mg GAE/g flour and FRAP = 2.24 mM FeSO_4_/g flour). The current study indicates that pigmented rice demonstrated higher values of phenolic compounds and antioxidant activity than white rice. These findings are similar to previous studies [16,40] that white rice had the lowest phenolic compounds and antioxidant activities when compared to pigmented rice. It has been shown that the bran of whole grain pigmented rice contains polyphenolic compounds including proanthocyanins, anthocyanins, and flavonoids which positively correlates with antioxidant capacity [41]. Therefore, it suggests that antioxidant activity of HN is partly attributed to its high content of polyphenolics and flavonoids. 

### 3.3. Thermal Properties

As shown in Table 1, the results showed that the gelatinization temperatures of HN including onset (T_o_), peak (T_p_) and conclusion (T_c_) were similar to those of HM. In general, differential scanning calorimetry (DSC) has been widely used to evaluate the thermal properties of starch, especially starch gelatinization. The endotherm for disordering of amylopectin crystallites is clarified by onset (T_o_), peak (T_p_) and conclusion (T_c_) temperatures. The values of gelatinization temperature of HN are the similar range to other rice cultivars such as brown rice [42] and glutinous rice [43]. However, HN flour had lower enthalpy change (ΔH) than HM flour, indicating that HN required low energy to convert the crystalline to an amorphous structure in starch granules [32]. In contrast, this effect consequently leads to less stability of crystals [22]. According to previous studies, the thermal parameters of starch are correlated with milling method, size, crystalline structure, amylose content, other chemical components, and starch composition (amylose to amylopectin ratio and phosphorous content) [44,45,46,47]. It has been shown that non-starch components in rice flour such as protein, ash, fiber and lipids cause a reduction of enthalpy for gelatinization [48]. Furthermore, the low enthalpy value of flour might be also attributed to low molecular weight and chain length distribution of amylopectin [49]. Further studies are required to clarify the starch structure of amylose and amylopectin in HN flour.

### 3.4. Swelling Power and Solubility

**Figure 4:**
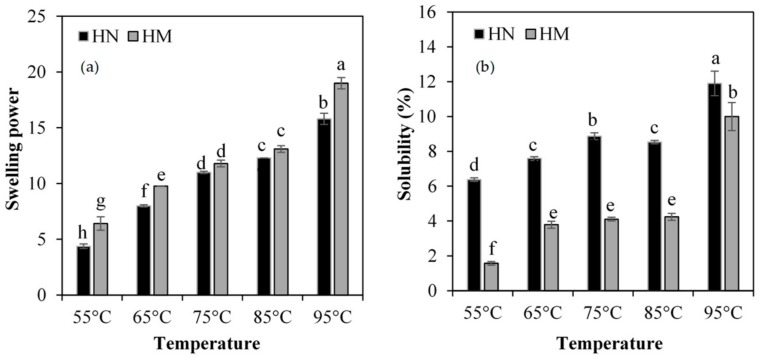
Swelling power (**a**) and solubility (**b**) of Hom Nil rice flour (HN) and Hom Mali 105 rice flour (HM). The results are expressed as mean ± SEM, *n* = 5. The different letters denote statistically significant differences in mean values. (*p* < 0.01) Mean values with the same superscript letters (a, b, c, d or e) were similar and no statistically significant differences were observed for these samples.

The result showed that swelling power (SP) and solubility (SB) of all rice flours significantly increased with an increase in temperature from 55 °C to 95 °C (Figure 4a,b). HN had lower swelling power in the range of 4.38 ± 0.2 g/g and 15.8 ± 0.5 g/g, and higher solubility in the range of 6.38 ± 0.1% and 11.9 ± 0.7% than HM (SP from 6.41 ± 0.6 to 19.0 ± 0.5 g/g and SB from 1.57 ± 0.1 to 10.0 ± 0.8%) at all temperatures.

During starch gelatinization, the hydrogen bonds between the hydroxyl groups in the double helices of starch molecules are disrupted and allowed the hydroxyl groups to form new hydrogen bonds with water molecules, resulting in the swelling of starch granules. This increases the accessibility of starch molecules to leach out from the inner part of the granules and then increases the solubility of starch molecules [47,50]. The results from the solubility suggest that HN had higher capacity to hold water than HM. Yu et al. suggest that the distribution of amylose and amylopectin in starch granules directly affect the solubility of starch [47]. Amylose maintains the structure of starch granule by predominant locating at the central region of the granules. Starch containing high amylose indicates more compact structure of starch granule that starch is more difficulty overflow outside the granules, leading to reduce solubility of starch. Therefore, the low amount of amylose could increase swelling power and solubility of starch due to a less rigid granular structure [47,51]. However, the current findings of the swelling power in HN are inconsistent with previous studies [47,51]. It is possible that other factors might affect the swelling power of HN flour. It has been shown that the swelling power may be influenced by not only amylose and amylopectin structure, degree of granulation, but also starch components, and other factors [37]. Li et al. revealed that various components of flours including starch, protein, dietary fiber, minerals, and phenolics may contribute to apparent difference in swelling power and solubility [46]. In addition, whole grain colored rice flour had lower swelling and higher solubility than polished white rice flour [37,46]. The presence of protein and lipid in rice flour granules may retard the water permeate into granules and this consequently induces the low level of swelling power [46,50]. Moreover, the particle size distribution of rice flour also affected swelling power and solubility. In addition, the low level of swelling power might be partly attributed to high concentration of phenolic compounds. Li et al. also demonstrated that these components could interact with the structure of starch, causing an increase in gelatinization temperature and a consequent reduction of swelling power [46].

### 3.5. Pasting Properties

**Figure 5:**
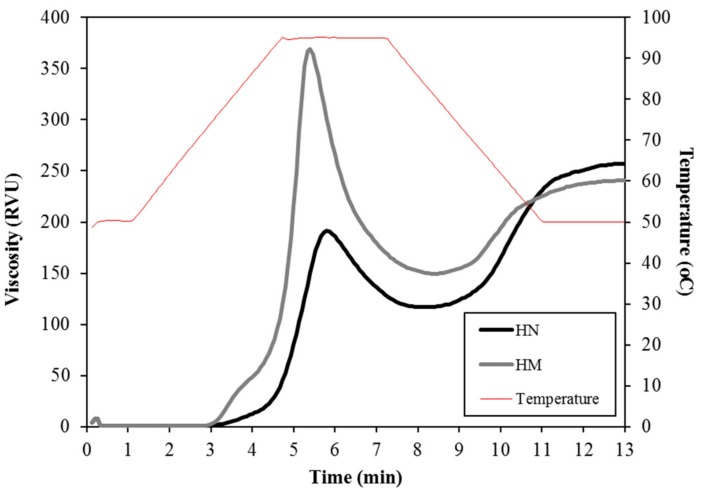
The mean pasting pattern of Hom Nil rice flour (HN) and Hom Mali 105 rice flour (HM).

The pasting characteristics of the flour are shown in Figure 5. The pasting profiles represent the pasting performance of flour during cooking and cooling, which is useful information to the starch or food processing industry. The peak time and pasting temperature of HN flour had higher value than that of HM flour (Table 2). The peak time represents the cooking time indicating the minimum temperature required to cook flour [52]. Pasting temperature provided the minimum of temperature needed for flour cooking [52]. Therefore, the results of this study indicate that HN flour required longer time and higher temperature for pasting process. In contrast, HM flour showed larger peak, trough and breakdown viscosity as compared to HN flour. Peak viscosity (PV) refers to the swelling extent or water-binding capacity of starch during heating process. After PV achieved, viscosity was dropped (breakdown viscosity), which indicates the degree of stability of swollen starch granules during cooking [37]. Trough viscosity is the viscosity reaching the minimum after cooling [52]. Previously, it has been shown that peak, trough and breakdown viscosity of rice flour increases with increasing amylose content [42]. The presence of high amylose content and high proportions of very long amylopectin branch chains provided high peak and breakdown viscosities of starch [53]. Therefore, the higher values of viscosity in HM with higher amylose content support these findings. In addition, larger final viscosity and setback were observed in HN. In cooling stage, the viscosity increased again, indicating by final viscosity and setback. Final viscosity implies the stability of cooked paste [52]. Setback indicates the retrogradation or staling of the flour paste [52]. Gani et al. reported that higher value of final viscosity and setback exhibited a greater tendency for retrogradation in food products during storage [54]. In the present study, HN had higher values of final viscosity and setback than HM, indicating that the high tendency to retrograde may occur in the products prepared from HN flour.

### 3.6. Characteristics of Rice Bread

Texture profiles and specific volume of HN and HM bread are shown in Table 3. It was found that HN bread had higher hardness and chewiness, whereas it had lower cohesiveness, adhesiveness and springiness than HM bread. However, there were no statistically significant differences in adhesiveness and specific volume of HN and HM bread. The obtained results can be explained by the physicochemical properties of rice flour. It has been shown that the textural characteristics of bread are largely affected by the structure and granule size of starch [55]. Subba et al. reported that the particle size of rice flour was negatively correlated with cohesiveness of products [56]. This report supports the findings that low cohesiveness of HN is attributed to its large particle size. In addition, hardness of bread crumb might be partly affected from the particle size of flour and gelatinization performance. In this study, HN had larger particle size with higher pasting temperature and time, resulting in more hardness of bread crumb. The bread properties are dependent on amylose content and gelatinization temperature of flour. It has shown that soft bread texture was obtained from rice flour with low amylose content (<20%) and low gelatinization temperature (<65 °C) [56]. In contrast, rice flour with high amylose content or high gelatinization temperature could produce the sandy texture of bread [57]. This statement supports by Aoki et al. indicating that low to medium amylose content in rice flour produces less hardness of bread than rice flour containing high amylose content [58]. However, the low-amylose rice flour showed asymmetry in the sides of the loaves curve [58]. Aoki et al. suggest that rice flour with medium amylose content and low gelatinization temperature rice could produce soft texture and good shape of bread [58]. The evidence did not support for the current findings of hardness in bread crumb. This might be related to other components in HN flour such as retaining of the rice bran. 

**Figure 6:**
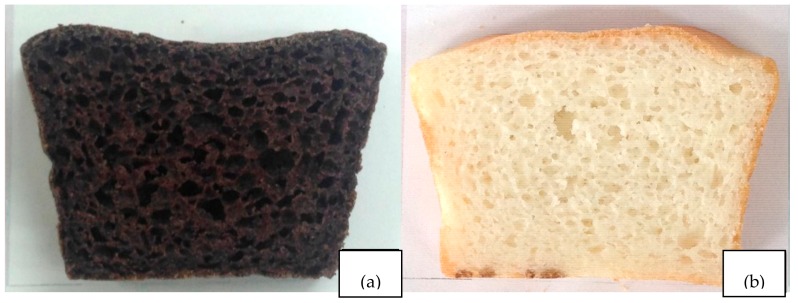
The cross section of gluten free bread from Hom Nil rice flour (**a**) and Hom Mali 105 rice flour (**b**).

The bread crumb appearance of gluten free bread from HN and HM are shown in Figure 6. HN bread was observed to have darker color than HM bread, relating to its content of anthocyanins. In addition, flattened surface was found in HN bread, indicating lower retention of air in bread batter due to larger particle size. However, the specific volume values of bread did not differ significantly. They were 2.37 ± 0.05 cm^3^/g for HN and 2.45 ± 0.00 cm^3^/g for HM, which consistent with a previous study reporting that preferable values ranged 2.35–2.41 cm^3^/g [22].

## 4. Conclusions

Hom Nil rice flour (HN) contained higher total polyphenolic and total anthocyanins as well as antioxidant activity than HM flour. The larger particle size and lower amount of amylose content was found in HN. In addition, HN flour demonstrated lower peak, trough and breakdown viscosity when compared to HM flour. Moreover, HN flour had lower swelling power and higher solubility than HM flour. Finally, the bread prepared by HN flour had higher value of hardness and chewiness, whereas it demonstrated lower value of cohesiveness, adhesiveness and springiness than the bread prepared from HM flour. These findings might be related to low amylose content and large particle size of HN flour. It suggests that HN could be an alternative gluten-free ingredient for bread products.

## Figures and Tables

**Table 1:** Thermal properties of Hom Nil rice flour (HN) and Hom Mali 105 rice flour (HM).

Rice Cultivar	Thermal Properties			
	T_o_ (°C)	T_p_ (°C)	T_c_ (°C)	ΔH (J/g)
HN	65.5 ± 0.3 ^a^	72.0 ± 0.7 ^a^	78.1 ± 0.9 ^a^	8.83 ± 0.1 ^a^
HM	65.3 ± 0.3 ^a^	71.1 ± 0.3 ^a^	77.0 ± 0.5 ^a^	9.47 ± 0.1 ^b^

The results are expressed as mean ± standard error of the mean (SEM), *n* = 3. The different letters denote statistically significant differences in mean values. (*p* < 0.01) Mean values with the same superscript letters (a or b) were similar and no statistically significant differences were observed for these samples.

**Table 2:** Pasting properties of Hom Nil rice flour (HN) and Hom Mali 105 rice flour (HM).

Rice Cultivar	Pasting Properties						
	Peak Viscosity (RVU)	Trough Viscosity (RVU)	Breakdown (RVU)	Final Viscosity (RVU)	Setback (RVU)	Peak Time (min)	Pasting Temperature (°C)
HN	191.5 ± 1.8 ^b^	116.8 ± 1.1 ^b^	74.6 ± 1.7 ^b^	257.2 ± 0.8 ^a^	140.3 ± 0.7 ^a^	5.82 ± 0.02 ^a^	87.8 ± 0.2 ^a^
HM	370.8 ± 1.8 ^a^	149.6 ± 1.6 ^a^	221.2 ± 2.4 ^a^	240.6 ± 1.4 ^b^	91.0 ± 0.2 ^b^	5.38 ± 0.02 ^b^	74.8 ± 0.2 ^b^

The results are expressed as mean ± standard error of the mean (SEM), *n* = 3. The different letters denote statistically significant differences in mean values. (*p* < 0.01) Mean values with the same superscript letters (a or b) were similar and no statistically significant differences were observed for these samples.

**Table 3:** Texture profile and specific volume of gluten free bread from Hom Nil rice flour (HN) and Hom Mali 105 rice flour (HM).

Characteristics	HN Bread	HM Bread
Texture profiles		
Hardness (N)	8.40 ± 0.28 ^a^	3.94 ± 0.10 ^b^
Cohesiveness	0.29 ± 0.01 ^b^	0.44 ± 0.01 ^b^
Chewiness (N.mm)	25.80 ± 1.3 ^a^	19.75 ± 0.90 ^b^
Springiness (mm)	10.43 ± 0.2 ^a^	11.40 ± 0.21 ^b^
Adhesiveness (N.mm)	0.03 ± 0.01 ^a^	0.05 ± 0.01 ^a^
Specific volume (cm^3^/g)	2.37 ± 0.05 ^a^	2.45 ± 0.00 ^a^

The results are expressed as mean ± standard error of the mean (SEM), *n* = 3 with 10 replicates per sample (texture analysis); *n* = 3 (specific volume). The different letters denote statistically significant differences in mean values. (*p* < 0.01) Mean values with the same superscript letters (a or b) were similar and no statistically significant differences were observed for these samples.
